# AMP-activated protein kinase agonist N_6_-(3-hydroxyphenyl)adenosine protects against fulminant hepatitis by suppressing inflammation and apoptosis

**DOI:** 10.1038/s41419-017-0118-0

**Published:** 2018-01-18

**Authors:** Jin Li, Bo Chen, Liping Zhong, Feng Gao, Haibo Zhu, Fengzhong Wang

**Affiliations:** 1 Institute of Food Science and Technology, Chinese Academy of Agricultural Sciences (CAAS), 100193 Beijing, China; 2 Institute of Medicinal Biotechnology, Chinese Academy of Medical Sciences & Peking Union Medical College, 100050 Beijing, China; 3 Life Science College of Tarim University, 843300 Xinjiang, China; 4 State Key Laboratory for Bioactive Substances and Functions of Natural Medicines and Beijing Key Laboratory of New Drug Mechanisms and Pharmacological Evaluation Study, Institute of Materia Medica, Chinese Academy of Medical Sciences & Peking Union Medical College, 100050 Beijing, China

## Abstract

Both AMP-activated protein kinase (AMPK) agonist and inhibitor have been reported to protect against fulminant hepatitis, implying that AMPK may play a complicated role in the development of fulminant hepatitis. In this study, we exploited whether the novel AMPK agonist N_6_-(3-hydroxyphenyl)adenosine (named as M1) exerted protective effects on fulminant hepatitis and whether its beneficial effects were AMPK-dependent. Results showed that intraperitoneal injection of M1 improved liver function, ameliorated liver injury and finally raised the survival rate in d-galactosamine/lipopolysaccharide (GalN/LPS)-treated mice. These beneficial effects of M1 may attribute to the suppression of pro-inflammatory cytokines production and the prevention of hepatocyte apoptosis. Furthermore, M1 pretreatment mitigated LPS-stimulated TLR4 expression and NFκB activation in murine peritoneal macrophages and prevented actinomycin D (Act D)/tumor necrosis factor α (TNFα)-induced apoptosis by promoting protective autophagy in primary hepatocytes. Additionally, M1-induced AMPK activation was responsible both for its anti-inflammatory action in macrophages and for its anti-apoptotic action in hepatocytes. To our surprise, compared with the control AMPKα1^lox/lox^/AMPKα2^lox/lox^ mice, liver-specific AMPKα1 knockout (AMPKα1_LS_^−/−^) mice were more sensitive to GalN/LPS administration but not AMPKα2_LS_^−/−^mice, and the beneficial effects of M1 on acute liver failure and the production of pro-inflammatory factors were dampened in AMPKα1_LS_^−/−^ mice. Therefore, our study may prove that M1 could be a promising therapeutic agent for fulminant hepatitis, and targeting AMPK may be useful therapeutically in the control of LPS-induced hepatotoxicity.

## Introduction

Viral infection, toxin, and drug poisoning, such as acetaminophen overdose are all the common etiologies contributing to liver disease. In western developed countries, acetaminophen overdose always causes acute liver failure, whereas viral hepatitis B-induced liver injury predominates in developing country^1^. Fulminant hepatitis has already been one of the dreaded clinical diseases with high mortality and poor prognosis. Although great progress has been made in acknowledging the molecular mechanism of fulminant hepatitis, there is still a long way to go, since liver transplantation may be the only method to cure the disease in the present. Fulminant hepatitis induced by lipopolysaccharide (LPS) and d-galactosamine (GalN) is a well-established experimental model, which is widely applicated to search for potential therapeutics. LPS, as the main inflammatory bacterial component, is usually used to trigger the activation of immune cells and initiate inflammation response, leading to multiple organ failure, especially hepatocyte dysfunction, while GalN is responsible for sensitizing to LPS lethality by inhibiting protein and RNA synthesis^2^. Other chemicals, such as actinomycin D (Act D) are also always used for sensitizing to LPS toxicity^3^. In an early phase, the production of pro-inflammation cytokines, especially tumor necrosis factor α (TNFα) are thought to be the most important step to cause liver injury, since these deleterious factors may trigger massive hepatocytes apoptosis and subsequent necrosis at the late stage^4^.

N_6_-(3-hydroxyphenyl)adenosine (named as M1) was first known as the main in vivo metabolite of O2′,O3′,O5′-tri-acetyl-N_6_-(3- hydroxyphenyl)adenosine (named as IMM-H007 or WS070117)^5^ and has been previously reported to alleviate airway inflammation in chronic obstructive pulmonary disease (COPD) induced by smoking cigarette plus LPS challenge^6^. The prototype drug IMM-H007 was cordycepin derivative and was discovered with several pharmacological actions, such as reducing blood lipids, improving liver steatosis and suppressing the formation of atherosclerotic plaque^7–9^. These beneficial effects were closely related to the activation of AMP-activated protein kinase (AMPK), which is a key enzyme in regulating energy metabolism and inflammation^10^. IMM-H007 or M1 was found to activate cellular AMPK by interacting with the γ subunit, but not by changing the cellular energy state^7^. In our previous study, cordycepin was found to protect against GalN/LPS-induced acute liver failure^11^. Therefore, we speculated that M1 might have the similar beneficial effects on fulminant hepatitis. Here, we report that intraperitoneal injection of M1 provide protection against liver injury caused by GalN/LPS. M1 pretreatment suppress GalN/LPS-induced hepatocytes apoptosis and the expression of inflammatory cytokines. Notably, M1 dampened LPS-stimulated TLR4 expression and NFκB activation in peritoneal macrophages and prevented Act D/TNFα-induced apoptosis by promoting autophagy in hepatocytes. Furthermore, M1-mediated AMPK activation was observed both in GalN/LPS-treated liver tissues and in LPS-stimulated macrophages. In vivo experiments in liver-specific AMPKα1 knockout mice revealed the pivotal role of AMPK in the development of fulminant hepatitis and the protective effects of M1 in further.

## Results

### M1 pretreatment attenuates GalN/LPS-induced hepatic failure

**Fig. 1:**
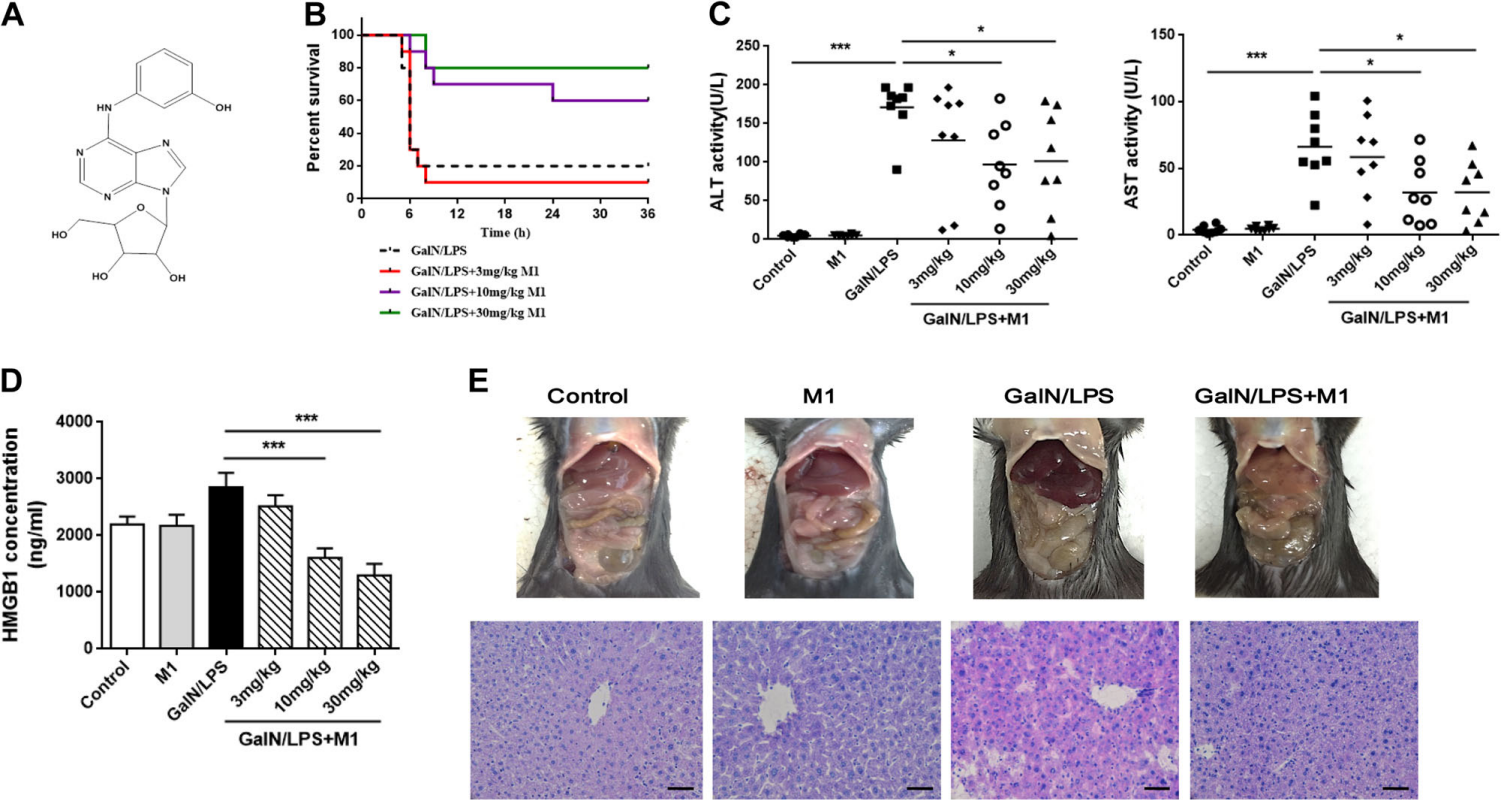
Intraperitoneal injection of M1 protects against GalN/LPS-induced fulminant hepatitis **a** The chemical structure of M1. **b** C57BL/6J mice were intraperitoneal injected with 3, 10, or 30 mg/kg M1 every 8 h one time for three times. GalN (400 mg/kg)/LPS (10 μg/kg) were then injected at 1 h after the last administration. The mortality rates were observed at various time points within 48 h (*n* = 10). C57BL/6J mice were intraperitoneal injected either with vehicle, 3, 10, or 30 mg/kg M1 every 8 h one time for three times. Blood and liver tissues were collected at 4 h after saline or GalN/LPS treatment. **c**, **d** Serum ALT, AST, and HMGB1 levels were assayed (*n* = 8). **e** Visibly different damaged livers and H&E staining analysis of liver sections are shown. Scale bars: 50 μm. Each value is mean ± S.E.M. **P < 0.01, ***P < 0.001

The cordycepin derivative M1 (Fig. 1a), has been previously reported to alleviate inflammation in lung and mitigate steatosis in the liver^6,7,12^. Here we tested whether it could improve GalN/LPS-induced hepatic injury. Analysis of survival rate in mice within 48 h after a lethal dose of GalN/LPS demonstrated that intraperitoneal injection with its metabolite M1 (3, 10, and 30 mg/kg) significantly raised the survival rate in a dose-dependent manner (Fig. 1b). Consistently, serum alanine aminotransferase (ALT), aspartate transaminase (AST), and high-mobility group box-1 (HMGB1) levels, which were widely accepted as markers of hepatic injury, increased at 4 h after GalN/LPS administration (Fig. 1c, d). Pretreatment with M1 (10 and 30 mg/kg) significantly decreased circulating ALT, AST, and HMGB1 levels. Histological grading of H&E sections and macroscopic examination confirmed attenuated liver injury induced by GalN/LPS in mice, which were injected with 30 mg/kg M1 in advance (Fig. 1e).

### M1 pretreatment suppresses GalN/LPS-induced hepatocyte apoptosis and inflammatory response

**Fig. 2:**
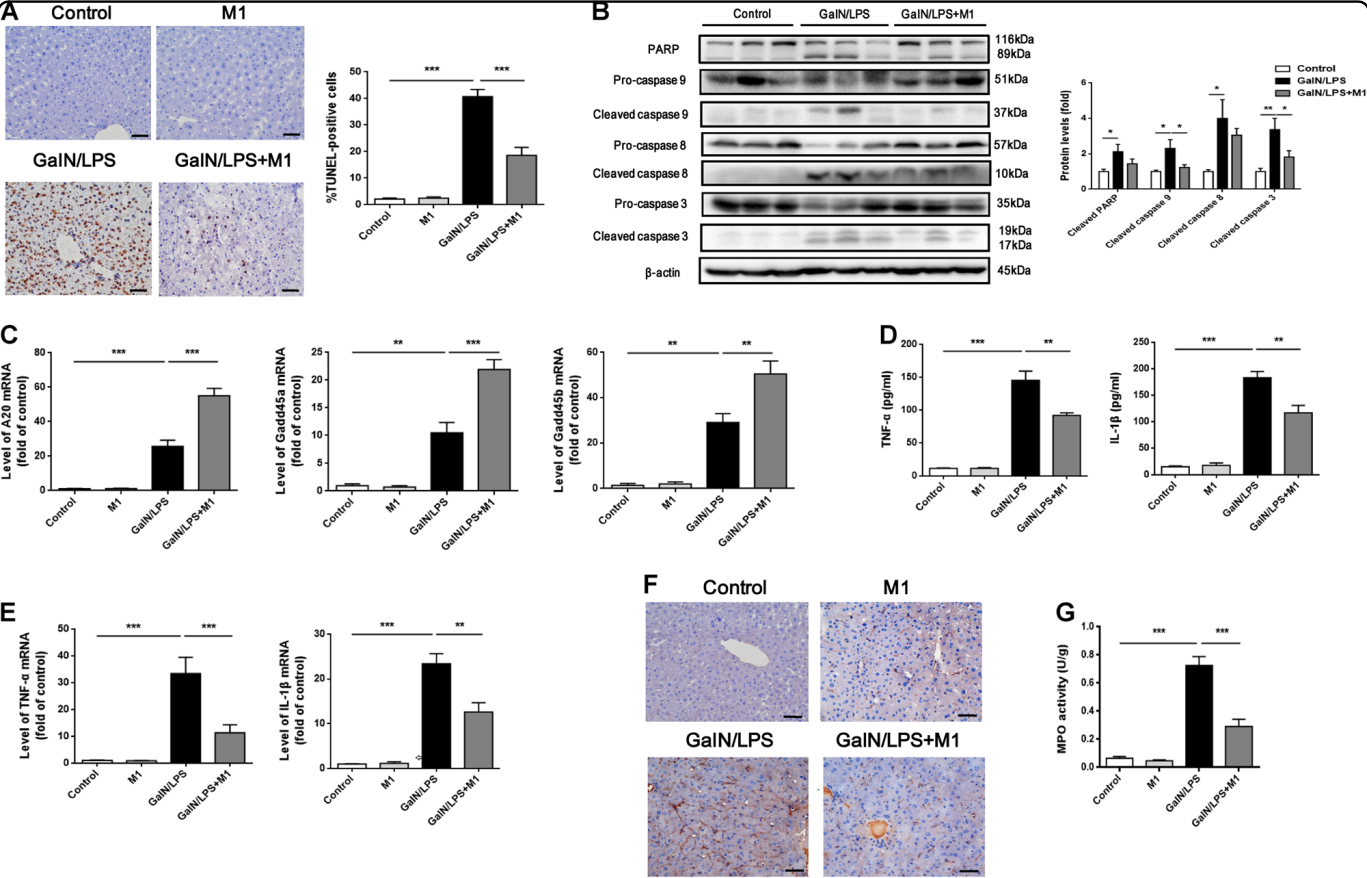
M1 suppresses hepatocytes apoptosis and inflammatory cytokines production caused by GalN/LPS C57BL/6J mice were intraperitoneal injected either with vehicle or 30 mg/kg M1 every 8 h one time for three times. Blood and liver tissues were collected at 4 h after GalN (400 mg/kg)/LPS (10 μg/kg) treatment. **a** Hepatocytes apoptosis were measured by TUNEL staining and the number of apoptotic cells were counted (*n* = 5). Scale bars: 50 μm. **b** The levels of cleaved caspase 3, caspase 8, caspase 9, and PARP were measured by western blot (*n* = 5). **c** The expression of hepatic genes Gadd45α, Gadd45β, and A20 were determined by QPCR (*n* = 3). **d** Serum TNFα and IL-1β levels were assayed using ELISA method (*n* = 8). **e** Hepatic TNFα and IL-1β mRNA expression were determined by QPCR (*n* = 4–6). **f** F4/80 staining of liver sections were conducted to indicate the filtration of macrophages. Scale bars: 50 μm. **g** Hepatic MPO activity was measured according to manufacturer’s instruction (*n* = 5–6). Each value is mean ± S.E.M. *P < 0.05, **P < 0.01, ***P < 0.001

To determine whether M1 could affect hepatocyte apoptosis, terminal deoxynucleotidyl transferase dUTP nick end labeling (TUNEL) stain and caspase activation were examined. As shown in Fig. 2a, the number of apoptotic hepatocytes was greatly increased (40.8 ± 2.6 per 100 nuclei) in GalN/LPS group, whereas in GalN/LPS plus 30 mg/kg M1 group, the number was significantly decreased (18.6 ± 3.0 per 100 nuclei). The levels of cleaved caspase 8, caspase 9, and caspase 3 were enhanced at 4 h after GalN/LPS administration. Similarly, the expression of cleaved poly-ADP-ribose polymerase (PARP), which is the substrate for caspases, was also increased. However, administrated with 30 mg/kg M1 markedly reduced the cleavage of caspase 3, caspase 8, caspase 9, and PARP (Fig. 2b). Additionally, the expression of hepatoprotective genes Gadd45α, Gadd45β, and A20 was increased in liver tissues of mice injected with GalN/LPS and M1 pretreatment further upregulated the expression of these genes (Fig. 2c). Therefore, M1 may attenuate GalN/LPS-induced fulminant hepatitis by inactivating apoptotic death signaling and promoting hepatocyte survival.

Serum levels of TNFα and interleukin-1β (IL-1β) were measured by enzyme linked immunosorbent assay (ELISA) to determine whether M1 could ameliorate inflammation. As predicted, TNFα and IL-1β production were remarkably enhanced at 4 h after GalN/LPS administration, whereas pretreatment with 30 mg/kg M1 significantly reduced TNFα and IL-1β expression (Fig. 2d). Realtime-PCR assay in hepatic tissues revealed that M1 pretreatment (30 mg/kg) diminished GalN/LPS-induced TNFα and IL-1β mRNA expression (Fig. 2e). Meanwhile, we found that GalN/LPS-induced increase of F4/80 expression and MPO activity, which indicate macrophage and neutrophil infiltration respectively, were attenuated by 30 mg/kg M1 administration (Fig. 2f, g).

### M1 inhibits LPS-induced TNFα production in macrophages through decreasing TLR4 expression and blocking NFκB activation

**Fig. 3:**
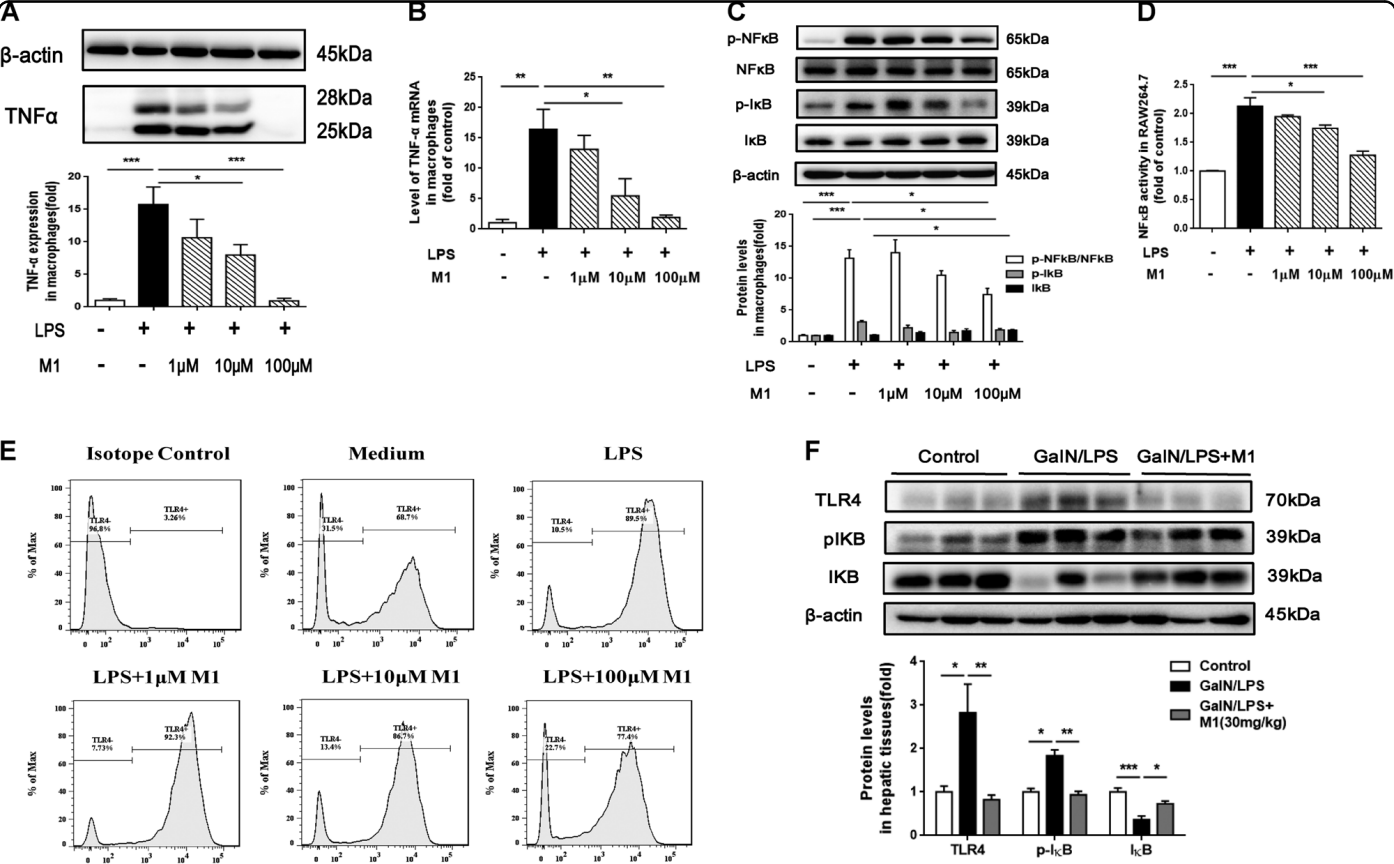
M1 prevents LPS-induced TNFα production in peritoneal macrophages via inhibiting TLR4/NFκB signal activation Murine peritoneal macrophages were treated with different concentration of M1 (1, 10, 100 μM) 1 h before the addition of 10 μg/ml LPS for 6 h. The protein level **a** and mRNA expression **b** of TNFα were assayed by western blot and QPCR, respectively (*n* = 3). **c** Then the relative expression of phosphor-NFκB, NFκB, phosphor-IκB, and IκB were quantified as well (*n* = 3). **d** NFκB activation were also determined by a luciferase reporter gene transfected in RAW264.7 cells (*n* = 3). **e** TLR4 expression on the surfaces of murine macrophages were detected by flow cytometry (*n* = 3). **f** Hepatic expression of TLR4, phosphor-IκB, and IκB were analyzed by western blot (*n* = 5). Each value is mean ± S.E.M. *P < 0.05, **P < 0.01, ***P < 0.001

It has been suggested that nuclear transcriptional factor NFκB activity is upregulated once LPS interacts with its receptor TLR4^13^. Therefore, TLR4 and NFκB signaling pathway may be implicated in the inhibitory effects of M1 on inflammatory cytokines production. In LPS-stimulated murine peritoneal macrophages, apparent increase both in protein level and mRNA expression of TNFα were observed, whereas M1 pretreatment (1, 10, and 100 μM) reduced TNFα protein and mRNA expression in a dose-dependent manner (Fig. 3a, b). Next, we determined the expression of phosphor-NFκB, NFκB, phosphor-IκB, and IκB in LPS-stimulated peritoneal macrophages. IκB is the inhibitor of NFκB and its phosphorylation contributes to its degradation^14^. The Ser536 phosphorylation of NFκB RelA/p65 has also been confirmed to be involved in its translocation from cytoplasm to nuclei^15^. As shown in Fig. 3c, the pretreatment of M1 dose-dependently prevented the enhanced expression of phosphor-NFκB/NFκB and phosphor-IκB induced by LPS, correspondingly increased the expression of IκB. In RAW 264.7 cells which were transfected with NFκB promoter luciferase reporter gene, we also found that M1 pretreatment significantly inhibited LPS-induced increase of NFκB activity (Fig. 3d). Meanwhile, pretreatment with M1 dose-dependently suppressed the expression of TLR4 on the cell surface of LPS-stimulated peritoneal macrophages as determined by flow cytometry (Fig. 3e). We then measured hepatic TLR4, phosphor-IκB, and IκB protein levels by western blots in further. As predicted, GalN/LPS-induced increased expression of TLR4 and phosphor-IκB, and decreased expression of IκB were countered by 30 mg/kg M1 pretreatment (Fig. 3f).

### M1 prevents Act D/TNFα-induced cell apoptosis by inducing protective autophagy

**Fig. 4:**
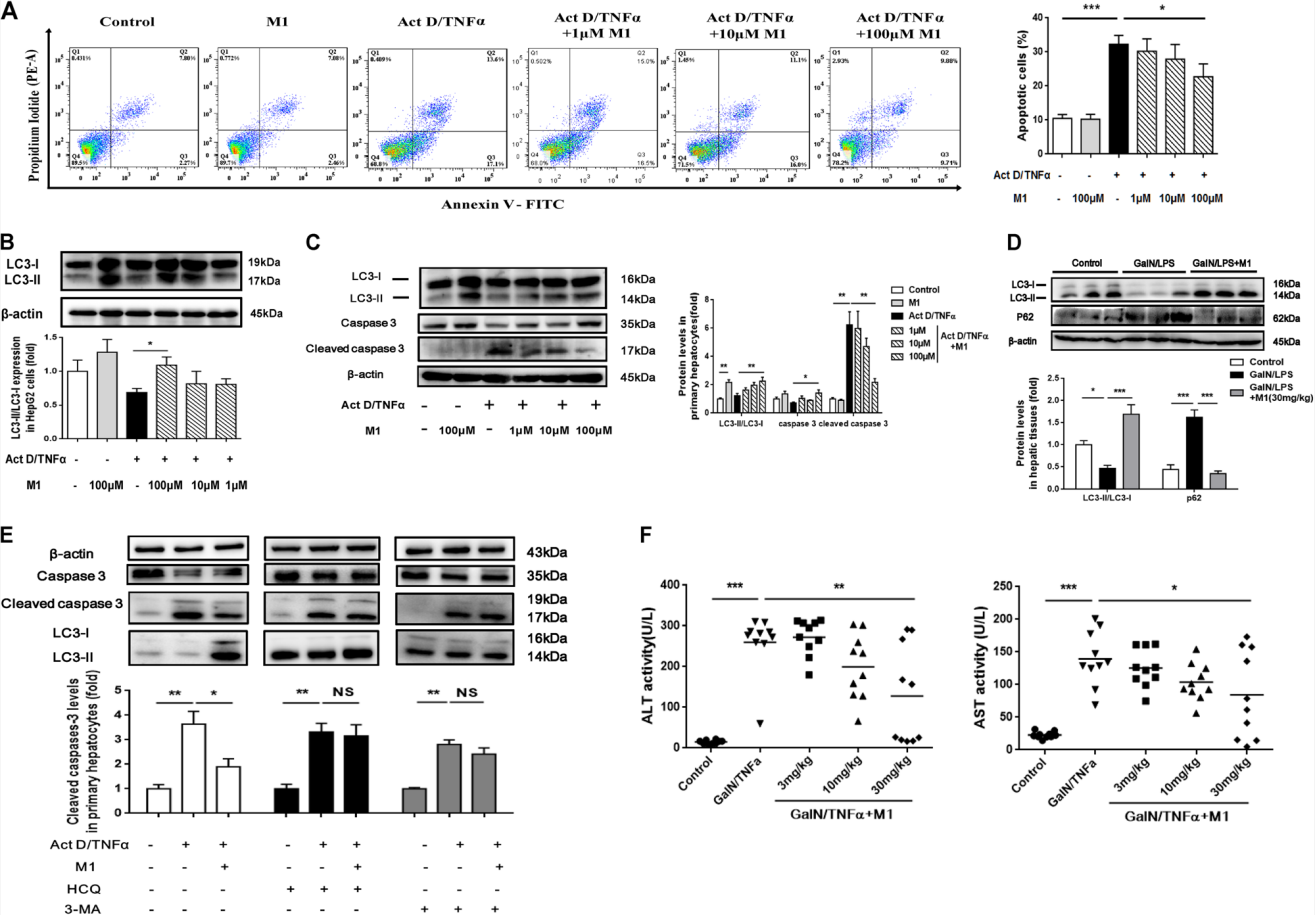
M1 inhibits Act D/TNFα-induced cell apoptosis via promoting autophagy HepG2 cells were treated with different concentrations of M1 (1, 10, and 100 μM) 1 h before the addition of 0.1 μg/ml Act D and 10 ng/ml TNFα for 12 h. **a** Apoptotic cells were measured by flow cytometry (*n* = 3). **b** Immunoblot analysis of LC-3 protein expression was conducted (*n* = 3). **c** Primary murine hepatocytes were treated with different concentrations of M1 (1, 10, and 100 μM) 1 h before the addition of 0.1 μg/ml Act D and 10 ng/ml TNFα for 12 h. Immunoblot analysis of LC-3, caspase 3, and cleaved caspase 3 protein expressions were conducted (*n* = 3). **d** C57BL/6J mice were intraperitoneal injected either with vehicle or 30 mg/kg M1 every 8 h one time for three times. Liver tissues were collected at 4 h after GalN (400 mg/kg)/LPS (10 μg/kg) treatment. Hepatic expression of LC3 and P62 were analyzed by western blot (*n* = 5). **e** Primary hepatocytes were treated with 100 μM M1 plus 75 μM HCQ or 2.5 mM 3-MA 1 h before the addition of 0.1 μg/ml Act D and 10 ng/ml TNFα for 12 h (*n* = 3). The expression of caspase 3, cleaved caspase 3, and LC3 were analyzed as indicated. **f** C57BL/6J mice were intraperitoneal injected with vehicle, 3 mg/kg, 10 mg/kg, or 30 mg/kg M1 every 8 h one time for three times. Blood samples were collected at 4 h after GalN (400 mg/kg)/TNFα (20 μg/kg) treatment. Serum ALT and AST levels were assayed (*n* = 10). Each value is mean ± S.E.M. *P < 0.05, **P < 0.01, ***P < 0.001

TNFα-induced cell apoptosis is thought to be the predominant event that contributes to liver injury in the model of GalN/LPS-induced fulminant hepatic failure^16^. Therefore, we tested whether M1 could protect TNFα-induced hepatocytes apoptosis. In the model of Act D/TNFα-stimulated HepG2 cells, large number of apoptotic cells appeared in Act D/TNFα-treated group whereas M1 pretreatment (100 μM) significantly reduced the number of apoptotic cells as measured by flow cytometry (Fig. 4a). Similarly, reduced caspase-3 cleavage was found in primary murine hepatocytes, which were incubated with 100 μM M1 before Act D/TNFα addition (Fig. 4c). It has been suggested that autophagy plays a vital role in TNFα cytotoxicity^17^. As predicted, Act D/TNFα caused little change in the expression of LC3-II/LC3-I, which is a hallmark of autophagy, whereas M1 pretreatment (100 μM) significantly increased the level of LC3-II in both HepG2 cells and primary hepatocytes (Fig. 4b, c), suggesting enhanced autophagic flux was induced by M1. Consistently, we also observed significant rescue of GalN/LPS-induced decreased autophagy in liver tissues of mice administrated with 30 mg/kg M1 (Fig. 4d). Next, hydroxychloroquine (HCQ) and 3-methyl adenine (3-MA), widely used as autophagy inhibitor, were applied to block M1-induced autophagy^18,19^. HCQ was believed to prevent lysosomal acidification and therefore prevent the degradation of LC3-II, whereas 3-MA was reported to curb the transformation from LC3-I to LC3-II due to its inhibitory effect on phosphoinositide 3-kinase (PI3K) activity. The administration of HCQ and 3-MA countered the anti-apoptotic effect of 100 μM M1 in primary hepatocytes (Fig. 4e). In mice injected with GalN/TNFα, pretreatment with 30 mg/kg M1 significantly prevented the increase in serum levels of ALT and AST (Fig. 4f). These results suggested that the pretreatment with M1 could prevent Act D/TNFα-induced cell death by inducing autophagy.

### AMPK is required for the anti-inflammatory effect and the anti-apoptotic effect of M1

**Fig. 5:**
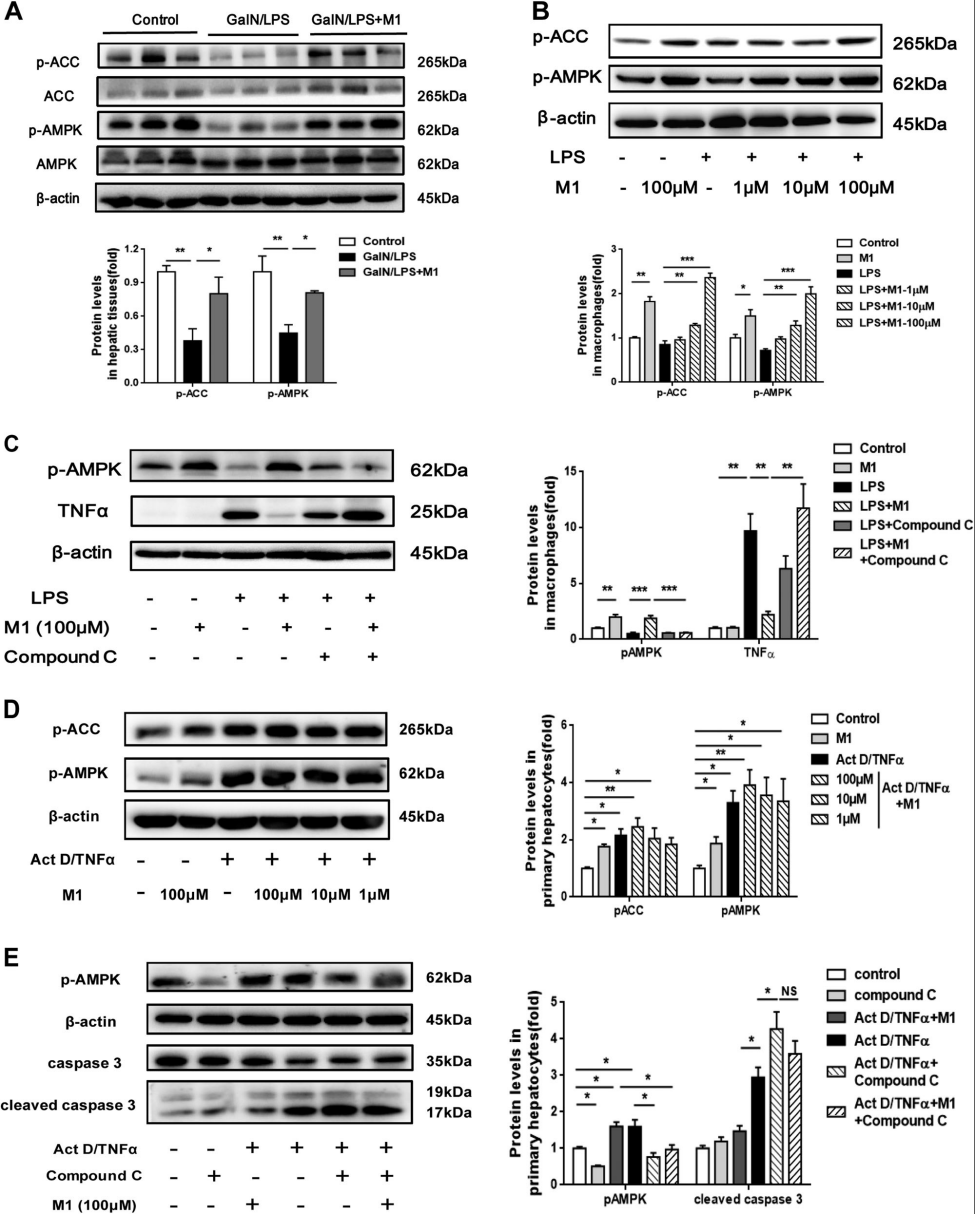
AMPK is required for M1 exerting  anti-inflammatory effect in macropahges and anti-apoptotic effect in  hepatocytes **a** C57BL/6J mice were intraperitoneal injected either with vehicle or 30 mg/kg M1 every 8 h one time for three times. Liver tissues were collected at 4 h after GalN (400 mg/kg)/LPS (10 μg/kg) treatment. The hepatic levels of phosphor-ACC and phosphor-AMPK were measured by western blot. Statistical analysis was conducted for five pieces of liver tissues in each group. **b** The expression of phosphor-AMPK and phosphor-ACC in primary macrophages treated with different concentrations of M1 (1, 10, 100 μM) 1 h before the addition of 10 μg/ml LPS for 6 h, were analyzed by immunoblotting (*n* = 3). **c** The decreased expression of TNFα mediated by 100 μM M1 in LPS-treated macrophages were rescued by pretreatment of 25 μM Compound C for 1 h. Immunoblot analysis of phosphor-ACC and phosphor-AMPK protein expression were conducted (*n* = 3). **d** Primary hepatocytes were treated with different concentrations of M1 (1, 10, and 100 μM) 1 h before the addition of 0.1 μg/ml Act D and 10 ng/ml TNFα for 12 h. Immunoblot analysis of phosphor-ACC and phosphor-AMPK protein expression were performed (*n* = 3). **e** Primary hepatocytes were pretreated with 25 μM Compound C for 1 h in the presence or absence of 100 μM M1 and then treated with 0.1 μg/ml Act D and 10 ng/ml TNFα for 12 h. The expression of phosphor-AMPK, caspase 3, and cleaved caspase 3 were conducted (*n* = 3). Each value is mean ± S.E.M. *P < 0.05, **P < 0.01, ***P < 0.001

It has been reported that IMM-H007 activated AMPK possibly by interacting with AMPKγ subunit^7^. Therefore, we speculated the beneficial effects of M1 in fulminant hepatitis might be associated with the modulation of AMPK activity. Indeed, we observed decreased AMPK activity in GalN/LPS-treated liver and M1 administration lead to the upregulation of AMPK activity, indicated by increased phosphorylation of downstream protein Acetyl-CoA carboxylase (ACC) (Fig. 5a). In LPS-stimulated murine macrophages, M1 caused a dose-dependent increase of phosphor-AMPK and phosphor-ACC (Fig. 5b). Pretreatment with Compound C, a specific AMPK inhibitor, countered M1-mediated decreased expression of TNFα (Fig. 5c), demonstrating that the attenuation of inflammation in response to M1 is dependent on AMPK activation. Additionally, we found upregulated AMPK activity induced by Act D/TNFα in primary hepatocytes and M1 administration caused a slight increase of AMPK activity when compared with Act D/TNFα group (Fig. 5d). Nevertheless, inhibition of AMPK by Compound C could not alleviate Act D/TNFα-induced cell apoptosis, but on the contrary, exacerbated the death and prevented the decrease expression in cleaved caspase 3 caused by M1 (Fig. 5e).

### AMPKα1 is required for the protective effects of M1 on GalN/LPS-induced fulminant hepatitis

**Fig. 6:**
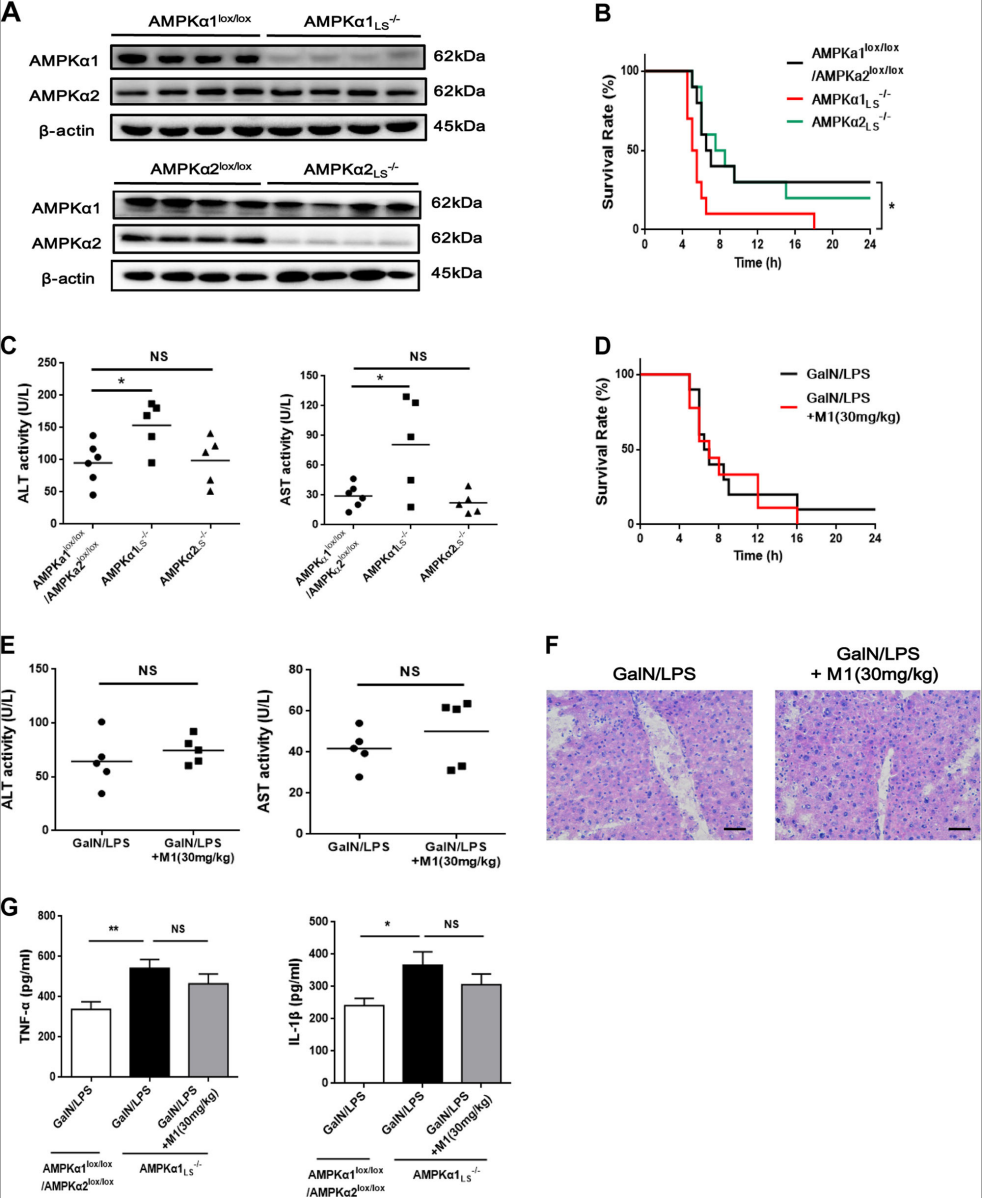
AMPK is responsible for M1 protecting against GalN/LPS-induced fulminant hepatitis **a** Representative western blots of liver tissues of AMPKα1^lox/lox^, AMPKα2^lox/lox^, AMPKα1_LS_^−/−^, and AMPKα2_LS_^−/−^ mice using antibody against AMPKα1, AMPKα2, and β-actin. AMPKα1^lox/lox^/AMPKα2^lox/lox^, AMPKα1_LS_^−/−^, and AMPKα2_LS_^−/−^ mice were injected with 400 mg/kg GalN plus 1 μg/kg LPS. **b** The mortality rates were observed at various time points within 48 h (*n* = 10). **c** Blood samples were harvested at 4 h after GalN/LPS administration. Serum ALT and AST levels were determined (*n* = 5–6). **d** AMPKα1_LS_^−/−^ mice were intraperitoneal injected with either vehicle or 30 mg/kg M1 every 8 h one time for three times. The mortality rates were observed at various time points within 48 h after injected with a lethal dose of GalN (400 mg/kg)/LPS (1 μg/kg) (*n* = 9–10). **e** Blood and liver samples were harvested at 4 h after GalN/LPS administration. Serum ALT and AST levels were determined (*n* = 5). **f** H&E staining analysis of liver sections are shown. Scale bars: 50 μm. **g** Serum TNFα and IL-1β levels were assayed using ELISA method (*n* = 8). Each value is mean ± S.E.M. *P < 0.05. NS, not significant

To confirm whether AMPK is necessary for the protective effect of M1 against GalN/LPS-induced fulminant hepatitis, liver-specific AMPKα1 and α2 knockout mice were generated. The AMPKα1 and α2 deletion were confirmed by western blots (Fig. 6a). Protein levels of hepatic AMPKα1 or α2 were failed to detect, whereas the expression of AMPKα2 or α1 was not changed, similar with the previous report^20^. Additionally, AMPKα1 or α2 defect only existed in liver tissue but not in other organs including heart, spleen, adipose tissue, skeletal muscle, and brain (data not shown). The mortality after GalN/LPS injection was greatly increased in AMPKα1_LS_^−/−^ mice when compared with the mortality of control AMPKα1^lox/lox^/AMPKα2^lox/lox^ mice, whereas there was no significant change in AMPKα2_LS_^−/−^ mice (Fig. 6b). Consistently, serum ALT and AST levels were upregulated in AMPKα1_LS_^−/−^ mice but not in AMPKα2_LS_^−/−^ mice (Fig. 6c). Furthermore, pretreatment with 30 mg/kg M1 could not alter the mortality and survival period in GalN/LPS-treated AMPKα1_LS_^−/−^ mice (Fig. 6d), and could not improve liver function and hepatocyte necrosis as well, indicated by the determination of serum ALT and AST activity and the observation of HE stains in liver sections (Fig. 6e, f). Meanwhile, M1 (30 mg/kg) administration could not lower the serum levels of TNFα and IL-1β in AMPKα1_LS_^−/−^ mice injected with GalN/LPS (Fig. 6g). These in vivo data suggest that hepatic AMPK is necessary for M1 protecting against fulminant hepatitis.

## Discussion

Fulminant hepatitis is a lethal disease with high mortality. The administration with a lethal dose of GalN/LPS to mice has become one of the most popular experimental models for screening potential therapeutic agents of fulminant hepatitis. GalN/LPS-induced liver injury largely depends on the production of TNFα, which will result in abundant apoptotic hepatocytes through the death receptor-dependent pathway^16^. Accumulating data suggested that both diminishing TNFα production at the early phase and inhibiting TNFα-mediated pro-apoptotic signal at the late phase are feasible and efficient strategies to prevent the occurrence of fulminant hepatitis^21,22^. Here we found that intraperitoneal injection of M1 dose-dependently protects against GalN/LPS-induced liver injury. M1 pretreatment decreased the circulating levels of pro-inflammatory cytokines in mice injected with GalN/LPS and dampened completely the expression of TNFα induced by LPS in peritoneal macrophages. Additionally, it was also found to diminish cell apoptosis in liver tissues of fulminant hepatitis and prevented Act D/TNFα-stimulated cell death both in primary hepatocytes and HepG2 cell lines. These data demonstrated that M1 exerted the protective effects on fulminant hepatitis through suppressing inflammation and apoptosis.

NFκB is one of the key transcription factors in the regulation of multiple inflammatory genes including cytokines and chemokines. The canonical NFκB signaling pathway is triggered once LPS interacts with its receptor TLR4 on the surface of hepatic macrophage^13^. Several natural compounds were reported to protect against acute liver failure by inhibiting NFκB-mediated pro-inflammatory cytokines production^23–25^. In this study, we observed that M1 exhibited negative impacts on NFκB activity and TLR4 expression in LPS-stimulated murine peritoneal macrophages. Consistently, M1 pretreatment diminished TLR4 expression and increased the expression of NFκB inhibitor IκB in GalN/LPS-treated liver tissues.

The general mechanism underlying hepatocyte apoptosis in fulminant hepatitis is the activation of extrinsic death-receptor-mediated apoptotic pathway by TNFα and Fas ligand. The following signal transport may involve caspase 8, caspase 9, and finally caspase 3 and PARP, which are effectors of apoptotic pathway^26^. Our results showed that M1 improved hepatic dysfunction induced by GalN/LPS partly via inhibiting hepatocyte apoptosis, indicated by reduced expression of cleaved caspase-8, caspase-9, caspase-3, and PARP in liver tissues of M1-treated GalN/LPS group. Consistently, we also observed M1 rescued Act D/TNFα-induced hepatocytes apoptosis, as shown in the results of flow cytometry and the assay of cleaved caspase-3. In mice injected with GalN/TNFα, pretreatment with 30 mg/kg M1 could significantly prevent the increased serum levels of ALT and AST, which confirmed the protective effects of M1 in TNFα-induced toxicity in vivo. It has been suggested that autophagy, a catabolic lysosomal degradation pathway, correlates well with acute liver injury, because genetic deletion of hepatic Atg5 or Atg7 contributes to cell death and finally liver dysfunction^27,28^. Accumulating evidence supports the idea that autophagy protects cells against apoptosis by suppressing caspase activation and apoptosis diminishes autophagy by degrading pivotal autophagy proteins^17,29,30^. We speculated that autophagy may be involved in the beneficial effects of M1 on Act D/TNFα-induced apoptosis. In this study, there was no significant change in the expression of LC3-II between vehicle-treated and Act D/TNFα-treated cells, whereas M1 pretreatment significantly induced autophagic flux in both primary hepatocytes and HepG2 cell line in a dose-dependent manner. Furthermore, the promoting effect of M1 on autophagy was dampened by autophagic flux inhibitor HCQ or 3-MA, and the inhibiting effect of M1 on apoptosis was blocked as well. However, more experiments should be performed including genetically inhibiting autophagy, such as siRNA against Atg7 or beclin1 to further confirm autophagy-dependent protective effect of M1 on ActD/TNFα-induced cell death.

In the study, we observed a slight downregulation of AMPK activity in LPS-stimulated peritoneal macrophages, similar with previous study^31^. Importantly, M1 upregulated AMPK activity and suppressed expression of TNFα in macrophages treated with LPS. Subsequently, Compound C, a selective AMPK inhibitor in competition with ATP-binding sites, was used to probe the relationship between M1-induced AMPK activation and TNFα production. Notably, the decreased expression of TNFα caused by M1 was dampened by Compound C, indicating that the anti-inflammatory effect of M1 is associated with AMPK activation. Both pharmacological activation of AMPK and overexpressing the constitute-active form of AMPK mutant have been confirmed to attenuate LPS-induced pro-inflammatory cytokines production in macrophages^21,31^. Additionally, we and other teams have verified that AMPK may cause an inactivation of NFκB by phosphorylating protein phosphatase 2A (PP2A) or Sirtuin 1^8,32,33^. AMPK-dependent TLR4 signal inhibition via the regulation of activating transcription factor 3 (ATF3) were confirmed in murine macrophages as well^34^. Thus, M1 may attenuate TNFα release through AMPK-mediated TLR4-NFκB inactivation.

In Act D/TNFα-treated hepatocytes, we observed significant upregulation of AMPK activity and M1 treatment caused a slight increase in AMPK activity compared with Act D/TNFα-treated hepatocytes. AMPK is such a sensitive kinase, that once its cellular energy status (AMP:ATP) gets altered, its activity will subsequently change. It has been reported that TNFα can induce the activation of PARP1, which can lead to ATP depletion^35^. Cells expressing kinase-dead mutant AMPKα1 are highly sensitive to TNFα-induced apoptosis^36^. Thus, AMPK activation seems to be an adaptive response to maintain energy supply. Therefore, AMPK inhibition cannot diminish apoptosis but exacerbated the death in our study. Furthermore, inhibition in AMPK activity by Compound C prevented the decrease in expression in cleaved caspase 3 caused by M1, suggesting that AMPK activation seemed to be beneficial both for cell survival against Act D/TNFα stimulus and the anti-apoptotic effect of M1.

It is confusing whether both AMPK agonists and inhibitors have been found to alleviate acute hepatic failure^21,37,38^, implying that AMPK may play a complicated role in the development of fulminant hepatitis. However, either AMPK agonists or inhibitors may exert the protective effects via AMPK-independent ways. Recently, a study conducted by Dr. Hu has regarded AMPK as a detrimental factor in acute liver failure by using Compound C in vivo^21^. They also found an incremental phosphorylation of hepatic AMPK in GalN/LPS-treated mice. But in this study, we observed a diminished AMPK activity. The deviation may originate in the absence of measuring p-ACC expression in their study. The phosphorylation of ACC provides a more clearer readout of AMPK activation than the phosphorylation of AMPK^39^. To elucidate the role of hepatocyte AMPK in fulminant hepatitis in further, we first employed liver-specific AMPKα1 knockout mice to confirm whether the protective effects of M1 on acute liver injury is AMPK-dependent. Compared with the control AMPKα1^lox/lox^/AMPKα2^lox/lox^ mice injected with GalN/LPS, AMPKα1_LS_^−/−^ mice was more sensitive to the stimulation of GalN/LPS, manifested by upregulated mortality and shortened survival period, demonstrating that AMPKα1 has an overall beneficial effect in fulminant hepatitis. Furthermore, pretreatment with M1 did not improve GalN/LPS-induced hepatic dysfunction in AMPKα1_LS_^−/−^ mice, demonstrating that hepatic AMPK is essential for M1 protecting against fulminant hepatitis in vivo. Additionally, we found in GalN/LPS-treated AMPKα1_LS_^−/−^ mice, serum levels of TNFα and IL1β greatly increased when compared with GalN/LPS-treated AMPKα1^lox/lox^/AMPKα2^lox/lox^ mice and M1 pretreatment did not cause any changes in their expression. Therefore, we speculated that the activation of hepatocyte AMPKα1 by M1 not only prevented TNFα-induced cell apoptosis but also alleviated systemic inflammation. It was reported that overexpression of AMPK or pharmacological activation in hepatocytes affected the production of inflammatory mediators in liver^40^. However, how hepatocyte AMPKα1 affect the production of pro-inflammatory factors in macrophages and then systemic inflammation remains unknown. More experiments should be done in GalN/LPS-treated AMPKα1_LS_^−/−^ mice.

Collectively, pretreatment with cordycepin derivative M1 was beneficial for fulminant hepatitis caused by GalN/LPS for its anti-inflammatory and anti-apoptotic action. M1 attenuated infiltration of monocytes/macrophages into liver, suppressed the release of pro-inflammatory cytokines, protected hepatocytes against apoptosis and necrosis, ameliorated liver injury and therefore raised the survival rate. In further, M1 dampened LPS-stimulated TLR4 expression and NFκB activation in peritoneal macrophages and prevented Act D/TNFα-induced apoptosis by promoting protective autophagy in hepatocytes. Most importantly, AMPK was implicated in the protective effects of M1 against acute liver injury and was indispensable for its anti-inflammatory effect and anti-apoptotic effect. Therefore, our study may provide M1 as a potential lead compound for fulminant hepatitis, and targeting AMPK may prove to be useful therapeutically in the control of acute liver injury.

## Materials and methods

### Treatment of animals

Fourteen-week-old male C57Bl/6J mice were purchased from Charles River Corporation (Beijing, China), given access to food and water ad libitum. All the experiments were conducted in accordance with the guidelines from the Animal Care and Committee of the Institute of Materia Medica, Chinese Academy of Medical Sciences and Peking Union Medical College (Beijing, China). After 1 week of adaptation, mice were intraperitoneally injected with M1 (3, 10, and 30 mg/kg; purity >98%; Institute of Materia Medica, Chinese Academy of Medical Sciences) dissolved in saline contained 5% DMSO every 8 h one time for three times. The corresponding control mice were administrated with an equivalent volume of vehicle. On the last day, mice were injected intraperitoneally with GalN (400 mg/kg; J&K Scientific, Beijing, China) and LPS (10 µg/kg; Escherichia coli, O111:B4; Sigma-Aldrich, St. Louis, USA) dissolved in saline at 1 h after M1 or vehicle administration. For survival experiments, 40 mice were randomly assigned into the following groups (each group, *n* = 10): (a) vehicle-treated GalN/LPS group, (b) 3 mg/kg M1-treated GalN/LPS group, (c) 10 mg/kg M1-treated GalN/LPS group, and (d) 30 mg/kg M1-treated GalN/LPS group. The survival rate of each group was monitored for 48 h. For biochemical analysis, mice were randomly divided into six experimental groups (each group, *n* = 8): (a) vehicle-treated control group, (b) 30 mg/kg M1-treated control group, (c) vehicle-treated GalN/LPS group, (d) 3 mg/kg M-treated GalN/LPS group, (e) 10 mg/kg M-treated GalN/LPS group, and (f) 30 mg/kg M-treated GalN/LPS group. Blood and liver samples were harvested at 4 h after GalN/LPS injection. For the in vivo experiments of GalN (400 mg/kg)/TNFα (20 µg/kg) toxicity, similar dosage regimen and animal grouping were adopted.

To generate liver-specific AMPKα1 and AMPKα2 knockout mice (AMPKα1_LS_^−/−^ and AMPKα2_LS_^−/−^), albumin-cre^+^ mice were crossed with liver-specific AMPKα1^lox/lox^ and AMPKα2^lox/lox^ mice (Jackson Lab, Bar Harbor, USA) on a C57BL/6 background as previously reported^20^. Genotyping and western blot were conducted to verify liver specific knockout of AMPK. Primers for the cre transgene are 5′-GATGGCAAACATACGCAAGGGAT-3′ and 5′-CTTGCGAACCTCATCACTCGTTGC-3′. Primers for the floxed AMPKα1 gene are 5′-CCCACCATCACTCCATCTCT-3′ and 5′-AGCCTGCTTGGCACACTTAT-3′. Primers for the floxed AMPKα2 gene are 5′-GCAGGCGAATTTCTGAGTTC-3′ and 5′-TCCCCTTGAACAAGCATACC-3′. Fourteen-week-old male mice were randomly divided into three groups (each group, *n* = 10): (a) GalN (400 mg/kg)/LPS (1 µg/kg)-treated AMPKα1^lox/lox^ or AMPKα2^lox/lox^ group, (b) GalN/LPS-treated AMPKα1_LS_^−/−^ group, and (c) GalN/LPS-treated AMPKα2_LS_^−/−^ group. For the experiments of M1 treatment in AMPKα1_LS_^−/−^ mice, similar dosage regimen was adopted as mentioned before. Sixteen-week-old male AMPKα1_LS_^−/−^ mice were randomly divided into two groups (each group, *n* = 9–10): (a) vehicle-treated GalN/LPS group and (b) 30 mg/kg M1-treated GalN/LPS group.

### Analysis of liver enzymes and serum cytokine levels

Blood samples drawn from the orbit and serum were separated. ALT and AST activities were analyzed using commercial kits from Nanjing Jiancheng Bioengineering Institute. Circulating levels of cytokines TNF-a, IL-1β, and HMGB1 were quantified using ELISA kits according to the manufacturer’s instructions (Cloud-clone Corporation, Houston, USA).

### Analysis of histopathology

The liver samples were frozen in OCT embedding medium and 6-µm-thick sections were prepared. Then the slices were stained with hematoxylin–eosin and the histological changes were evaluated in nonconsecutive, randomly chosen histological fields. Apoptotic cells were detected by TUNEL method using an in situ apoptosis detection kit (TaKaRa Co., Shiga, Japan). The number of TUNEL-positive cells was recorded. At least three tissue sections in each group were analyzed.

### Immunohistochemistry

After treatment with hydrogen peroxide, frozen sections were incubated with rabbit anti-F4/80 antibody (diluted at 1:200, catalog number: sc-26643, Santa Cruz Biotechnology, Dallas, USA) for 1 h at 37 °C. The sections were then incubated in secondary antibody (catalog number: SAP-9100, Sino Biological, Beijing, China) following three washes. Prior to counterstaining with hematoxylin, these sections were colored using diaminobenzidine (DAB) kit. Negative controls were established using rabbit IgG instead of primary antibodies.

### Cell culture

Male C57BL/6J mice were used for the preparation of primary macrophages and primary hepatocytes according to the methods as described before^41,42^. Macrophages were incubated in RPIM1640 with 10% fetal bovine serum (FBS). Hepatocytes were incubated in Williams’ Medium E supplemented with 5% FBS, 100 nM insulin, 100 nM dexamethasone, 2 mM l-glutamine, 100 IU/ml penicillin, and 100 mg/ml streptomycin. After 4 h, non-adherent cells were removed and fresh media were added. The adherent cells were then cultured in the same medium without serum overnight before treatment. HepG2 cells were obtained from the cell culture center of Peking Union Medical College (PUMC, China) and were grown in DMEM plus 10% FBS. All cells were maintained at 37 °C in a 5% CO_2_/air environment.

### Quantitative real-time polymerase chain reaction (qRT-PCR) analysis

Total RNA was extracted from liver tissues by Trizol reagent (Sigma-Aldrich, St. Louis, USA). cDNAs were synthesized using reverse transcription kit (Toyobo, Shanghai, China) and then was amplified in PCRs with SYBR Green Supermix kit (Toyobo, Shanghai, China). The sequences of primers are as follows: mouse TNFα, forward 5′- GCCTCTTCTCATTCCTGCTTGT-3′, reverse 5′-TTGAGATCCATGCCGTTG-3′; mouse IL-1β, forward 5′-TTGACGGACCCCAAAAGAT-3′, reverse 5′-GATGATCTGAGTGTGAGGGTCTG-3′; mouse Gadd45α, forward 5′-GGACTCGCACTTGCAATATGAC-3′, reverse 5′-GTGTCCATCCTTTCGGTCTTCT-3′; mouse Gadd45β, forward 5′-TGCATCATGACCCTGGAAGAG-3′, reverse 5′-ACCGCCTGCATCTTCTGAAC-3′; mouse A20 forward 5′-CTGAAAACCAATGGTGATGGAAA-3′, reverse 5′-TGAACACCCCACATGTACTGACA-3′; mouse GAPDH, forward 5′-GCCTGGAGAAACCTGCCAAGTAT-3′, reverse 5′- GATGCCTGCTTCACCACCTTC-3′. The reactions were performed on Applied Biosystems Prism 7900HT. GAPDH served as internal normalization control.

### Western blot analysis

Proteins were prepared from liver tissues in RIPA lysis buffer with protease inhibitors and phosphatase inhibitors cocktail (Roche, Switzerland). Then SDS-PAGE was conducted and bands were transferred to PVDF membrane, incubating with primary antibodies against toll-like receptor 4 (TLR4), microtubule-associated protein light chain 3 (LC3), phosphor-IκB and phosphor-ACC (diluted at 1:1000, catalog number: ab45104, ab48394, ab12135 and ab68191, respectively, Abcam, Cambridge, UK), phosphor-NFκB, NFκB, IκB, caspase-3, caspase-8, caspase-9, poly-(ADP-ribose) polymerase (PARP), and phosphor-AMPK (diluted at 1:1000, catalog number: 3033, 8242, 4814, 9665, 4790, 9508, 9532, and 2535, respectively, Cell Signaling Technology, Boston, USA). Corresponding secondary antibodies (diluted at 1:5000, catalog numbers: ZDR-5306 or ZDR-5307, Sino Biological, Beijing, China) were added and bands were visualized with enhanced chemiluminescence reagents (Thermo Fisher Scientific, Waltham, USA). Signals were normalized to that of β-actin (diluted at 1:5000, catalog number: 4967, Cell Signaling Technology, Boston, USA).

### Flow cytometry analysis

Murine peritoneal macrophages were stained with PE-labeled anti-TLR4 antibody (catalog number: ab45104, Abcam, Cambridge, UK). After washed three times with PBS, TLR4 levels of cell surface were subsequently analyzed by flow cytometry (FACSVerse, BD, USA). The apoptotic cells were assayed by Annexin V-FITC method with flow cytometry according to manufacturer’s instruction (Transgen Biotechnology, Beijing, China).

### Luciferase reporter assay

RAW264.7 cells (Peking Union Medical College, China) were transiently transfected with both pNFκB-Luc plasmid and pRL-TK *Renilla* plasmid by using lipofectamine 3000 (Invitrogen, USA). After 24 h, cells were incubated with different concentration of M1 (1, 10, and 100 μM) followed by 10 μg/ml LPS. Cell lysis was determined for luciferase activity according to the luciferase assay reagent kit (Beyotime Biotechnology, Shanghai, China). The activity was divided by the activity of Renilla control reporter to normalize the transfection efficiency.

### Data analysis

Survival data were analyzed by the Kaplan–Meier curve and log-rank test. All other data were analyzed by one-way analysis of variance (ANOVA), and the Bonferroni test was used for post-hoc comparisons. The differences between each group were considered statistically significant at a *p*-value < 0.05. The results are presented as mean ± S.E.M.

## References

[1] Zimmermann HW, Trautwein C, Tacke F (2012). Functional role of monocytes and macrophages for the inflammatory response in acute liver injury. Front. Physiol..

[2] Silverstein R (2004). D-galactosamine lethality model: scope and limitations. J. Endotoxin Res..

[3] Ni HM (2016). Caspase inhibition prevents tumor necrosis factor-α-induced apoptosis and promotes necrotic cell death in mouse hepatocytes in vivo and in vitro. Am. J. Pathol..

[4] An J (2012). TAT-apoptosis repressor with caspase recruitment domain protein transduction rescues mice from fulminant liver failure. Hepatology.

[5] Liu Y, He J, Abliz Z, Zhu H (2011). In vitro stability and metabolism of O2’, O3’, O5’-tri-acetyl-N6-(3-hydroxylaniline) adenosine in rat, dog and human plasma: chemical hydrolysis and role of plasma esterases. Xenobiotica.

[6] Cao SH (2015). [Effect of WS070117M1 on chronic obstructive pulmonary disease in mice and the underling mechanisms of anti-inflammation]. Yao xue xue bao=Acta Pharm. Sin..

[7] Guo P (2012). The adenosine derivative 2’,3’,5’-tri-O-acetyl-N6-(3-hydroxylaniline) adenosine activates AMPK and regulates lipid metabolism in vitro and in vivo. Life Sci..

[8] Chen B, Li J, Zhu H (2016). AMP-activated protein kinase attenuates oxLDL uptake in macrophages through PP2A/NF-kappaB/LOX-1 pathway. Vasc. Pharmacol..

[9] Lian Z (2011). A novel AMPK activator, WS070117, improves lipid metabolism discords in hamsters and HepG2 cells. Lipids Health Dis..

[10] Li J, Zhong L, Wang F, Zhu H (2017). Dissecting the role of AMP-activated protein kinase in human diseases. Acta Pharm. Sin. B.

[11] Li J, Zhong L, Zhu H, Wang F (2017). The protective effect of cordycepin on D-galactosamine/lipopolysaccharide-induced acute liver injury. Mediat. Inflamm..

[12] Sun Y, Lian Z, Jiang C, Wang Y, Zhu H (2012). Beneficial metabolic effects of 2’,3’,5’-tri-acetyl-N6- (3-hydroxylaniline) adenosine in the liver and plasma of hyperlipidemic hamsters. PLoS One.

[13] Takeuchi O, Akira S (2010). Pattern recognition receptors and inflammation. Cell.

[14] Yamamoto Y, Gaynor RB (2004). IkappaB kinases: key regulators of the NF-kappaB pathway. Trends Biochem. Sci..

[15] Chen PJ (2016). Honokiol suppresses TNF-alpha-induced neutrophil adhesion on cerebral endothelial cells by disrupting polyubiquitination and degradation of IkappaBalpha. Sci. Rep..

[16] Nowak M (2000). LPS-induced liver injury in D-galactosamine-sensitized mice requires secreted TNF-alpha and the TNF-p55 receptor. Am. J. Physiol. Regul. Integr. Comp. Physiol..

[17] Amir M (2013). Inhibition of hepatocyte autophagy increases tumor necrosis factor-dependent liver injury by promoting caspase-8 activation. Cell Death Differ..

[18] Wang H (2016). Erianin induces G2/M-phase arrest, apoptosis, and autophagy via the ROS/JNK signaling pathway in human osteosarcoma cells in vitro and in vivo. Cell Death Dis..

[19] Liu L (2016). Everolimus enhances cellular cytotoxicity of lapatinib via the eukaryotic elongation factor-2 kinase pathway in nasopharyngeal carcinoma cells. OncoTargets Ther..

[20] Andreelli F (2006). Liver adenosine monophosphate-activated kinase-alpha2 catalytic subunit is a key target for the control of hepatic glucose production by adiponectin and leptin but not insulin. Endocrinology.

[21] Hu K (2017). Endogenous AMPK acts as a detrimental factor in fulminant hepatitis via potentiating JNK-dependent hepatocyte apoptosis. Cell Death Dis..

[22] Yan T (2016). Glycyrrhizin protects against acetaminophen-induced acute liver injury via alleviating tumor necrosis factor alpha-mediated apoptosis. Drug Metab. Dispos..

[23] Ma L (2015). Sesamin ameliorates lipopolysaccharide/d-galactosamine-induced fulminant hepatic failure by suppression of Toll-like receptor 4 signaling in mice. Biochem. Biophys. Res. Commun..

[24] Xie YL (2017). Curcumin attenuates lipopolysaccharide/d-galactosamine-induced acute liver injury by activating Nrf2 nuclear translocation and inhibiting NF-kB activation. Biomed. Pharmacother..

[25] Zhang W (2016). Dioscin alleviates dimethylnitrosamine-induced acute liver injury through regulating apoptosis, oxidative stress and inflammation. Environ. Toxicol. Pharmacol..

[26] Luedde T, Kaplowitz N, Schwabe RF (2014). Cell death and cell death responses in liver disease: mechanisms and clinical relevance. Gastroenterology.

[27] Ni HM (2012). Liver-specific loss of Atg5 causes persistent activation of Nrf2 and protects against acetaminophen-induced liver injury. Toxicol. Sci..

[28] Komatsu M (2005). Impairment of starvation-induced and constitutive autophagy in Atg7-deficient mice. J. Cell. Biol..

[29] Ni HM, Bockus A, Boggess N, Jaeschke H, Ding WX (2012). Activation of autophagy protects against acetaminophen-induced hepatotoxicity. Hepatology.

[30] Li H (2011). Following cytochrome c release, autophagy is inhibited during chemotherapy-induced apoptosis by caspase 8-mediated cleavage of Beclin 1. Cancer Res..

[31] Sag D, Carling D, Stout RD, Suttles J (2008). Adenosine 5’-monophosphate-activated protein kinase promotes macrophage polarization to an anti-inflammatory functional phenotype. J. Immunol..

[32] Kim KY (2009). Adiponectin-activated AMPK stimulates dephosphorylation of AKT through protein phosphatase 2A activation. Cancer Res..

[33] Yang Z, Kahn BB, Shi H, Xue BZ (2010). Macrophage alpha1 AMP-activated protein kinase (alpha1AMPK) antagonizes fatty acid-induced inflammation through SIRT1. J. Biol. Chem..

[34] Liu X (2016). The citrus flavonoid naringenin confers protection in a murine endotoxaemia model through AMPK-ATF3-dependent negative regulation of the TLR4 signalling pathway. Sci. Rep..

[35] Huang Q, Shen HM (2009). To die or to live: the dual role of poly(ADP-ribose) polymerase-1 in autophagy and necrosis under oxidative stress and DNA damage. Autophagy.

[36] Kim SY (2012). AMP-activated protein kinase-alpha1 as an activating kinase of TGF-beta-activated kinase 1 has a key role in inflammatory signals. Cell Death Dis..

[37] Guo Y (2014). AMPK inhibition blocks ROS-NFkappaB signaling and attenuates endotoxemia-induced liver injury. PLoS One.

[38] Cai L (2015). AMPK dependent protective effects of metformin on tumor necrosis factor-induced apoptotic liver injury. Biochem. Biophys. Res. Commun..

[39] Park SH (2002). Phosphorylation-activity relationships of AMPK and acetyl-CoA carboxylase in muscle. J. Appl. Physiol..

[40] Buler M (2012). Energy-sensing factors coactivator peroxisome proliferator-activated receptor γ coactivator 1-α (PGC-1α) and AMP-activated protein kinase control expression of inflammatory mediators in liver: induction of interleukin 1 receptor antagonist*. J. Biol. Chem..

[41] Ray, A., Dittel, B. N. Isolation of mouse peritoneal cavity cells. *J. Vis. Exp*. (2010). 10.3791/1488 PMC3152216 20110936

[42] Shen, L., Hillebrand, A., Wang, D. Q., Liu, M. Isolation and primary culture of rat hepatic cells. *J. Vis. Exp.* (2012). 10.3791/3917 PMC3471302 22781923

